# Modified Vaccinia Virus Ankara Triggers Type I IFN Production in Murine Conventional Dendritic Cells via a cGAS/STING-Mediated Cytosolic DNA-Sensing Pathway

**DOI:** 10.1371/journal.ppat.1003989

**Published:** 2014-04-17

**Authors:** Peihong Dai, Weiyi Wang, Hua Cao, Francesca Avogadri, Lianpan Dai, Ingo Drexler, Johanna A. Joyce, Xiao-Dong Li, Zhijian Chen, Taha Merghoub, Stewart Shuman, Liang Deng

**Affiliations:** 1 Dermatology Service, Department of Medicine, Memorial Sloan Kettering Cancer Center, New York, New York, United States of America; 2 Molecular Biology Program, Memorial Sloan Kettering Cancer Center, New York, New York, United States of America; 3 Immunology Program, Memorial Sloan Kettering Cancer Center, New York, New York, United States of America; 4 Institute for Virology, Düsseldorf University Hospital, Heinrich-Heine-University, Düsseldorf, Germany; 5 Cancer Biology & Genetics Program, Memorial Sloan Kettering Cancer Center, New York, New York, United States of America; 6 Department of Molecular Biology, University of Texas, Southwestern Medical Center, Dallas, Texas, United States of America; 7 Lucille Castori Center for Microbes, Inflammation and Cancer, Memorial Sloan Kettering Cancer Center, New York, New York, United States of America; University of Alberta, Canada

## Abstract

Modified vaccinia virus Ankara (MVA) is an attenuated poxvirus that has been engineered as a vaccine against infectious agents and cancers. Our goal is to understand how MVA modulates innate immunity in dendritic cells (DCs), which can provide insights to vaccine design. In this study, using murine bone marrow-derived dendritic cells, we assessed type I interferon (IFN) gene induction and protein secretion in response to MVA infection. We report that MVA infection elicits the production of type I IFN in murine conventional dendritic cells (cDCs), but not in plasmacytoid dendritic cells (pDCs). Transcription factors IRF3 (IFN regulatory factor 3) and IRF7, and the positive feedback loop mediated by IFNAR1 (IFN alpha/beta receptor 1), are required for the induction. MVA induction of type I IFN is fully dependent on STING (stimulator of IFN genes) and the newly discovered cytosolic DNA sensor cGAS (cyclic guanosine monophosphate-adenosine monophosphate synthase). MVA infection of cDCs triggers phosphorylation of TBK1 (Tank-binding kinase 1) and IRF3, which is abolished in the absence of cGAS and STING. Furthermore, intravenous delivery of MVA induces type I IFN in wild-type mice, but not in mice lacking STING or IRF3. Treatment of cDCs with inhibitors of endosomal and lysosomal acidification or the lysosomal enzyme Cathepsin B attenuated MVA-induced type I IFN production, indicating that lysosomal enzymatic processing of virions is important for MVA sensing. Taken together, our results demonstrate a critical role of the cGAS/STING-mediated cytosolic DNA-sensing pathway for type I IFN induction in cDCs by MVA. We present evidence that vaccinia virulence factors E3 and N1 inhibit the activation of IRF3 and the induction of IFNB gene in MVA-infected cDCs.

## Author Summary

Modified vaccinia virus Ankara (MVA) is an attenuated vaccinia strain with large deletions of the parental genome that render it non-replicative in mammalian cells. MVA is a safe and effective vaccine against both smallpox and monkeypox. MVA has been investigated as a vaccine vector for infectious diseases and cancers. Dendritic cells (DCs) play important roles in innate and adaptive immunity. A better understanding of how MVA is detected by innate immune sensors in DCs would guide the development of more effective MVA-based vaccines. We report our findings that MVA infection induces the production of type I interferon (IFN) in conventional dendritic cells via a cytosolic DNA-sensing pathway mediated by the newly discovered DNA sensor cGAS, its adaptor STING, and transcription factors IRF3 and IRF7. By contrast, wild-type vaccinia virus fails to activate this pathway. Furthermore, we show that vaccinia virulence factors E3 and N1 play inhibitory roles in the cytosolic DNA-sensing pathway.

## Introduction

Poxviruses are large cytoplasmic DNA viruses that cause human and veterinary diseases. Variola virus (the causative agent of smallpox) and monkeypox virus are important human pathogens [1]–[3]. Modified vaccinia virus Ankara (MVA) is an attenuated vaccinia virus that was developed through serial passaging in chicken embryonic fibroblasts. MVA has a 31-kb deletion of the parental vaccinia genome and was used successfully as a vaccine during the WHO-sponsored smallpox eradication campaign [4]–[6]. MVA has been investigated intensively as a vaccine vector against HIV, tuberculosis and malaria, as well as cancers [7]–[12].

Dendritic cells are the sentinels of the immune system. They can be mainly classified into two subtypes: conventional dendritic cells (cDCs) and plasmacytoid dendritic cells (pDCs). cDCs are professional antigen-presenting cells that can be activated via Toll-like receptors (TLRs), RIG-I-like receptors, and cytosolic DNA-sensing pathways [13], [14]. pDCs are potent type I interferon (IFN) producing cells that sense viral infections via TLR7, TLR8, and TLR9, and their adaptor MyD88 [15]. Delineating the innate immune responses of dendritic cells to MVA infection could guide vaccine design using MVA-based vectors.

We reported previously that wild-type vaccinia (WT VAC) infection of epidermal cDCs fails to induce the production of type I IFN and attenuates innate immune responses to lipopolysaccharide (LPS) or poly(I∶C) [16]. Infection of human or murine pDCs with live WT VAC also fails to induce type I IFN production, whereas infection with heat-inactivated vaccinia (Heat-VAC, by incubating at 55°C for 1 h) induces TLR7/MyD88-dependent type I IFN production [17], [18]. These results indicate that WT VAC produces inhibitor(s) to block poxviral sensing in cDCs and pDCs.

MVA has deletions or truncations of several intracellular immunomodulatory genes including K1L, N1L, and A52R, which have been implicated in regulating innate immune responses, especially the NF-κB signaling pathway [19]–[24]. Vaccinia N1 is a 14-kDa cytosolic protein that contributes to virulence in murine infection models [25], [26]. In addition to its role in inhibiting the NF-κB pathway, N1 also attenuates IRF3 activation [21]. On the other hand, MVA retains the E3L gene encoding a bifunctional Z-DNA/dsRNA binding protein, a key vaccinia virulence factor [27]–[35]. It has been shown that MVA infection of human monocyte-derived dendritic cells causes DC activation [36]. Waibler et al. [37] reported that MVA infection of murine Flt3L-DC triggered a TLR-independent type I IFN response. In addition, MVA infection of human macrophages triggers type I IFN and pro-inflammatory cytokines and chemokines via a TLR2/TLR6/MyD88 and MDA5/MAVS-dependent pathways [38].

In this paper, we report that MVA infection of murine pDCs fails to induce type I IFN induction. By contrast, MVA infection of cDCs triggers type I IFN production. This induction in cDCs is dependent on STING (stimulator of interferon genes; an adaptor for the cytosolic DNA-sensing pathway), cGAS (cyclic GMP-AMP synthase; a recently discovered cytosolic DNA sensor), transcription factors IRF3 and IRF7, and the IFN receptor IFNAR1. We find that IFN production in MVA-infected cDCs is independent of RNA-sensing pathways mediated by MDA5, MAVS, TLR3, or TRIF, and is modestly affected by the absence of the TLR9/MyD88 endosomal DNA-sensing pathway. We present evidence that vaccinia E3 and N1 proteins play inhibitory roles in the cGAS/STING/IRF3-dependent cytosolic DNA-sensing pathway. Type I IFN serves as an important link between innate and adaptive immunity. Therefore, the identification of the cytosolic DNA-sensing pathway mediated by cGAS/STING/IRF3 for type I IFN induction by MVA in cDCs has implications for MVA-based vaccine design to improve its immunogenicity and efficacy.

## Results

### MVA induces type I IFN production in murine cDCs, but not in pDCs

**Figure 1:**
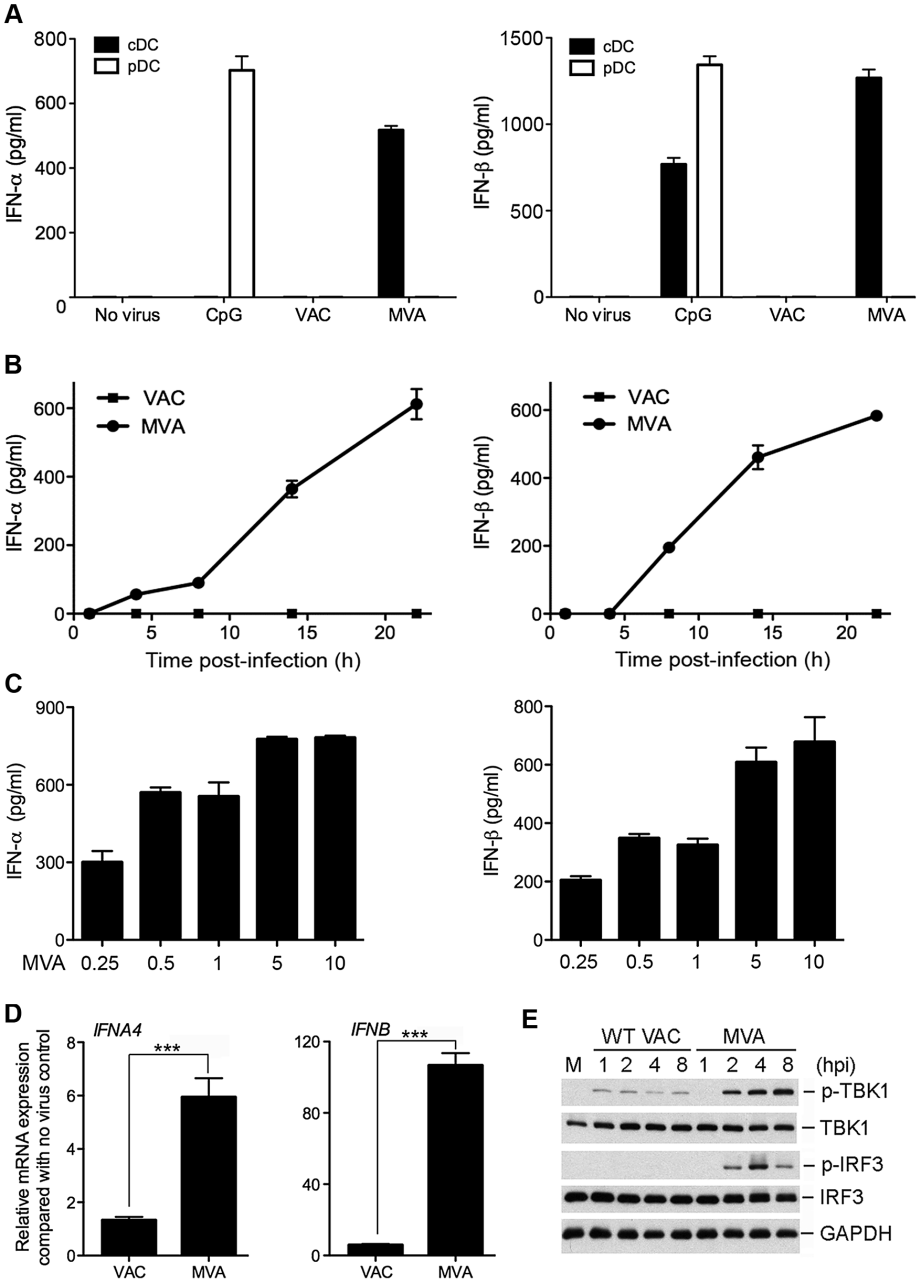
MVA induces type I IFN production in conventional dendritic cells (cDCs). (A) Murine pDCs and cDCs were purified from Flt3L-BMDCs using FACS. 2×10^5^ pDCs (CD11c^+^B220^+^PDCA-1^+^) and 1×10^6^ cDCs (CD11c^+^B220^−^PDCA-1^−^) were either stimulated with CpG (at a final concentration of 10 µg/ml) or infected with either WT VAC or MVA at a MOI of 10. Supernatants were collected at 22 h post infection. The concentrations of IFN-α and IFN-β were determined by ELISA. Data are means ± SEM (n = 6). A representative experiment is shown, repeated at least twice. (B) GM-CSF-BMDCs (1×10^6^) were infected with WT VAC or MVA at a MOI of 10. Supernatants were collected at 1, 4, 8, 14, and 22 h post infection. The concentrations of IFN-α and IFN-β were determined by ELISA. Data are means ± SEM (n = 3). A representative experiment is shown, repeated once. (C) GM-CSF-BMDCs (1×10^6^) were infected with MVA at a MOI of 0.25, 0.5, 1, 5, or 10. Supernatants were collected at 22 h post infection. The concentrations of IFN-α and IFN-β were determined by ELISA. Data are means ± SEM (n = 3). A representative experiment is shown, repeated once. (D) GM-CSF-BMDCs (1×10^6^) were infected with MVA or WT VAC at a MOI of 10. Cells were collected at 6 h post infection. Real-time PCR analysis of IFNA4 and IFNB mRNAs were performed. Data are means ± SEM (n = 3). A representative experiment is shown, repeated twice. ***, *p*<0.001; comparisons were made between MVA and WT VAC infected cells. (E) GM-CSF-BMDCs (1×10^6^) were infected with MVA or WT VAC at a MOI of 10. Cells were collected at 1, 2, 4, and 8 h post infection. Western blot analysis was performed using anti-phospho-TBK1, anti-TBK1, anti-phosphoserine-396 of IRF3, and anti-IRF3. Glyceraldehyde 3-Phosphate Dehydrogenase (GAPDH) was used as a loading control. “hpi”, hours post infection. “M”, mock infection control.

Waibler et al. [37] reported that MVA induces TLR-independent type I IFN responses in murine bone marrow-derived dendritic cells (BMDCs). Consistent with their report, we also observed that MVA infection of GM-CSF-cultured BMDCs or Flt3L-cultured BMDCs induces type I IFN secretion (data not shown). GM-CSF (Granulocyte/macrophage colony stimulating factor) is important for the development of CD11c^+^B220^−^PDCA1^−^ cDCs. Flt3L (fms-like tyrosine kinase-3 ligand) is critical for the commitment and differentiation of hematopoietic progenitors to both pDC and cDC populations [39], [40]. To determine which DC subtype is responsible for the production of type I IFN in response to MVA infection, we enriched pDCs and cDCs to 98% purity from Flt3L-cultured BMDCs (Flt3L-DCs) using FACS. 2×10^5^ pDCs and 1×10^6^ cDCs were stimulated with CpG or infected with either WT VAC or MVA at a multiplicity of infection (MOI) of 10. Supernatants were collected at 22 h post infection. The levels of IFN-α and IFN-β were determined by ELISA. We found that MVA infection induced the secretion of IFN-α/β from cDCs, but not from pDCs (Figure 1A). Treatment of cells with the TLR9 agonist CpG induced IFN-α and β production from pDCs, but only IFN-β from cDCs (Figure 1A). WT VAC infection failed to induce type I IFN production in either pDCs or cDCs (Figure 1A).

To assess the time course of induction of type I IFN secretion by MVA-infected cDCs, we performed kinetic analysis using GM-CSF-cultured BMDCs (cDCs), which demonstrated that IFN-α and IFN-β proteins were detected by ELISA at 8 h post-infection with MVA and continued to accumulate up to 24 h post-infection (Figure 1B).

To test whether MVA induces type I IFN production in cDCs in a dose-dependent manner, we infected cDCs with MVA at increasing MOIs from 0.25 to 10. We found that MVA induced IFN-α and IFN-β production even at a low MOI of 0.25. IFN production was increased with higher doses of MVA, which reached the highest level at a MOI of 5 and 10 (Figure 1C). We used MOI of 10 for MVA infection in the rest of the *in vitro* infection experiments reported in this paper.

### MVA infection up-regulates type I IFN mRNAs in murine cDCs

To test whether WT VAC or MVA infection of cDCs affects type I IFN gene expression, we performed quantitative real-time PCR analysis of RNA isolated from GM-CSF-cultured cDCs infected with WT VAC or MVA at 6 h post-infection. Mock-infection controls were also included. We observed that MVA infection of cDCs increased IFNA4 and IFNB mRNA levels by 6-fold and 105-fold, respectively, when compared with untreated cells. By contrast, infection with WT VAC increased IFNA4 and IFNB mRNA levels by 2-fold and 6-fold, respectively (Figure 1D). These results indicate that MVA is a stronger inducer of IFNA4 and IFNB gene expression than WT VAC (*p*<0.001).

### MVA infection of cDCs induces the phosphorylation of TBK1 and IRF3

We hypothesize that the differences we observed in IFN gene expression between MVA and WT VAC might be related to their abilities to activate transcription factor IRF3. IRF3 is a cytoplasmic protein expressed constitutively in many cell types. Upon virus infection, IRF3 is phosphorylated at multiple serine and threonine residues near the C-terminus. Phosphorylated IRF3 then translocates to the nucleus and activates IFN gene transcription [41]. We performed Western blot analysis of MVA- or WT VAC-infected cDCs, and found that MVA infection triggered IRF3 phosphorylation (which peaks at 4 h post infection), whereas WT VAC infection fails to do so. We also observed that MVA infection resulted in much higher levels of phosphorylation of TBK1 than WT VAC (Figure 1E), indicating that WT VAC might encode inhibitor(s) that interfere with phosphorylation of TBK1 and IRF3.

### MVA-induced type I IFN production in murine cDCs is dependent on IRF3/IRF7/IFNAR1

**Figure 2:**
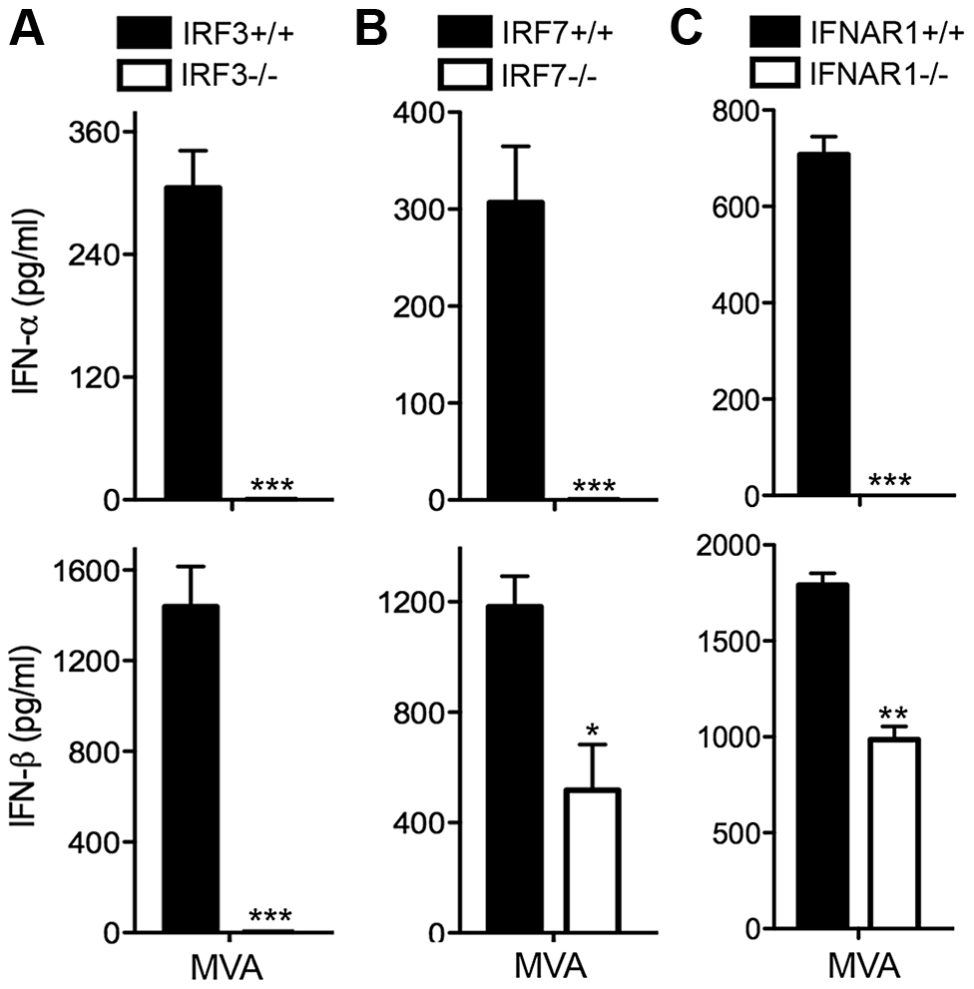
Transcription factors IRF3/IRF7 and the type I IFN positive feedback loop mediated by IFNAR1 are required for the induction of type I IFN in murine cDCs by MVA. GM-CSF-BMDCs were generated from IRF3^−/−^ (A), IRF7^−/−^ (B), IFNAR1^−/−^ (C) mice, and their age-matched WT controls. Cells (1×10^6^) were stimulated with CpG or infected with MVA at a MOI of 10. Supernatants were collected 22 h later. The concentrations of IFN-α and IFN-β were determined by ELISA. Data are means ± SEM (n = 3). A representative experiment is shown, repeated twice. *, *p*<0.05; **, *p*<0.01; ***, *p*<0.001; comparisons were made between WT cells and various knockout cells as indicated.

Similar to IRF3, the transcription factor IRF7 is another key regulator of type I IFN induction and is critical for host defense against virus infections [42], [43]. Using cDCs generated from IRF3^−/−^ and age-matched WT control mice, we found that MVA-induced IFN-α/β secretion was abolished in IRF3 deficient cDCs (Figure 2A). IRF7^−/−^ cells fail to produce IFN-α in response to MVA infection. IFN-β induction was reduced by 57% in MVA-infected IRF7^−/−^ cells (Figure 2B). To assess whether the type I IFN positive feedback loop mediated by IFNAR1 is required for the induction of IFN, we infected IFNAR1^−/−^ cDCs and WT controls with MVA at a MOI of 10. We found that IFN-α induction by MVA was abolished in IFNAR1^−/−^ cells, whereas IFN-β induction by MVA was reduced by 45% in IFNAR1^−/−^ cells compared with WT controls (Figure 2C). These results indicate that: (i) IRF3 is the critical transcription factor for MVA-induced type I IFN production, and (ii) IRF7 and IFNAR1 play roles in amplifying type I IFN signaling induced by MVA infection.

### Contributions of the endosomal DNA-sensing pathway via TLR9/MyD88 to innate immune sensing of MVA in murine cDCs

**Figure 3:**
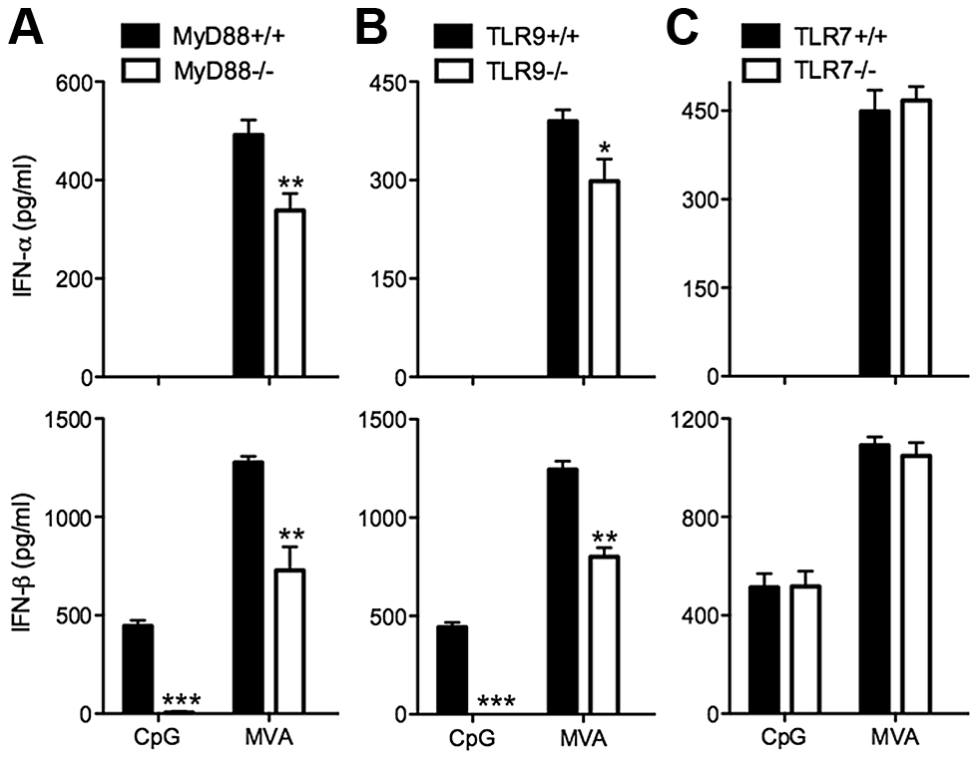
TLR9 and MyD88 contribute to the induction of type I IFN in cDCs by MVA. GM-CSF-BMDCs were generated from MyD88^−/−^ (A), TLR9^−/−^ (B), TLR7^−/−^ (C) mice, and their age-matched WT controls. Cells (1×10^6^) were either stimulated with CpG or infected with MVA at a MOI of 10. Supernatants were collected 22 h later. The concentrations of IFN-α and IFN-β were determined by ELISA. Data are means ± SEM (n = 6). The combined results of three independently performed experiments are shown. *, *p*<0.05; **, *p*<0.01; ***, *p*<0.001; comparisons were made between WT cells and various knockout cells as indicated.

To test whether TLR7, TLR9 and MyD88 are involved in MVA induction of type I IFN in murine cDCs, we generated cDCs from TLR7^−/−^, TLR9^−/−^, and MyD88^−/−^ mice or their age-matched WT controls. Cells were either treated with TLR9 agonist CpG or infected with MVA at a MOI of 10. Control experiments show that induction of IFN-β by CpG was abolished in MyD88^−/−^ and TLR9^−/−^ murine cDCs, but was not affected in TLR7^−/−^ cDCs (Figure 3A, 3B and 3C). By contrast, MVA-induced production of IFN-α and IFN-β was reduced by 32% and 43% in MyD88^−/−^ cDCs, and by 23% and 37% in TLR9^−/−^ cDCs, respectively, but was not affected in TLR7^−/−^ cDCs (Figure 3A, 3B and 3C). These results indicate that the endosomal TLR9/MyD88 pathway plays a minor role in MVA-sensing in cDCs.

### MVA induction of type I IFN in murine cDCs does not require TLR3, TRIF, MDA5 or MAVS

We assessed the contributions of other known nucleic acid-sensing pathways to the induction of type I IFN by MVA. MAVS (mitochondrial antiviral signaling protein) is an adaptor for the cytosolic RNA sensors RIG-I and MDA5 [44]. TRIF (TIR-domain-containing adapter-inducing interferon-β) is an adaptor for the endosomal TLR3 that senses extracellular dsRNA [45], [46] and an adaptor for cytosolic RNA sensors [47]. We found that TLR3, TRIF, MDA5, and MAVS deficient cells secreted similar amounts of type I IFN compared to cDCs from age-matched WT control mice in response to MVA infection (Figure S1A, B, C and D). Taken together, these results indicate MVA-induced type I IFN production by infected cDCs does not require previously known RNA-sensing mechanisms.

### MVA-induced type I IFN induction is dependent on STING

**Figure 4:**
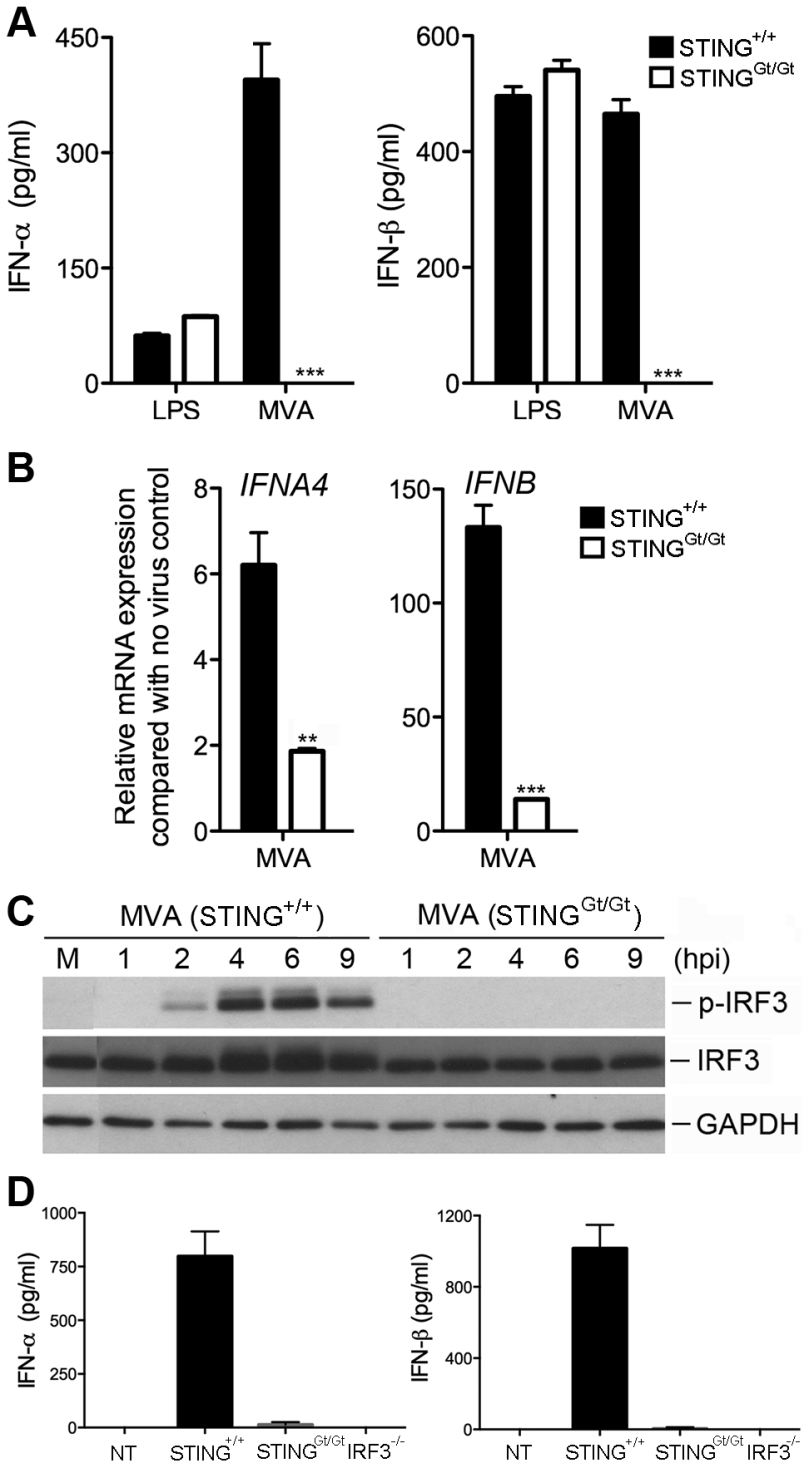
STING is required for the induction of type I IFN and IRF3 phosphorylation by MVA in BMDCs. GM-CSF-BMDCs were generated from WT mice (Sting^+/+^) and the N-ethyl-N-nitrosourea (ENU)-induced *Goldenticket* (*Gt*) mutant mice (Sting^Gt/Gt^) harboring a single nucleotide variant of Sting resulting in a functionally null allele. (A) Cells (1×10^6^) were either stimulated with LPS or infected with MVA at a MOI of 10. Supernatants were collected 22 h later. The concentrations of IFN-α and IFN-β were determined by ELISA. Data are means ± SEM (n = 3). Results shown are representative of three experiments. ***, *p*<0.001; comparisons were made between Sting^+/+^ and Sting^Gt/Gt^ as indicated. (B) Cells (1×10^6^) were infected with MVA at a MOI of 10. Cells were collected at 6 h post infection. Real-time PCR analysis of IFNA4 and IFNB mRNAs were performed. Data are means ± SEM (n = 3). A representative experiment is shown, repeated twice. **, *p*<0.01; ***, *p*<0.001; comparisons were made between Sting^+/+^ and Sting^Gt/Gt^ as indicated. (C) Western blot analysis of BMDCs from Sting^+/+^ and Sting^Gt/Gt^ infected with MVA at a MOI of 10, or mock infected. Whole-cell lysates were prepared. Equal amount of proteins were subjected to SDS-PAGE and immunoblotting with anti-phospho-TBK1, anti-TBK1, anti-phosphoserine-396 of IRF3, and anti-IRF3. GAPDH was used as a loading control. “hpi”, hours post infection. “M”, mock infection control. (D) Sting^Gt/Gt^, IRF3^−/−^ and age-matched WT C57B/6 control mice were infected with MVA (2×10^7^ pfu) via intravenous inoculation. Serum levels of IFN-α and IFN-β were determined by ELISA. Data are means ± SD. Results shown are representative of two independent experiments.

STING is an endoplasmic reticulum-associated protein essential for type I IFN induction in response to intracellular DNA or DNA pathogens including bacteria and DNA viruses [48]–[53]. STING is also important for the development of CD8^+^ T cell responses after vaccination with vaccinia virus expressing ovalbumin [50]. To test whether STING is required for type I IFN induction in cDCs by MVA, we generated cDCs from the N-ethyl-N-nitrosourea (ENU)-induced *Goldenticket* (*Gt*) mutant mice (Sting^Gt/Gt^) harboring a single nucleotide variant of *Sting* resulting in a functionally null allele [54]. cDCs from age-matched WT mice were used as a control. Cells were either infected with MVA at a MOI of 10 or treated with lipopolysaccharide (LPS). MVA induction of IFN-α/β was abolished in Sting^Gt/Gt^ cells, whereas LPS-induced IFN-α/β production was not affected (Figure 4A). Induction of IFNA4 mRNA by MVA was reduced from 6-fold in WT cells to 2-fold in Sting^Gt/Gt^ cells, whereas induction of IFNB mRNA by MVA was reduced from 133 fold in WT cells to 14 fold in Sting^Gt/Gt^ cells (Figure 4B). Western blot analysis demonstrated that MVA-induced IRF3 phosphorylation peaked at 4 and 6 h post infection in WT cDCs and was absent in Sting^Gt/Gt^ cDCs (Figure 4C). Together, these results demonstrate that STING is essential for MVA-induced type I IFN production and IRF3 phosphorylation in cDCs.

### MVA triggers type I IFN production *in vivo* in a STING/IRF3-dependent manner

The kinetics of type I IFN induction by MVA have been reported by Waibler et al. [55]. C57B/6 mice were infected with 1×10^7^ pfu of MVA through intravenous (i.v.) inoculation and serum was collected at 6, 12, 18, and 24 h. They found that MVA induced highest IFN-α production at 6 h post infection. We performed i.v. inoculation of purified MVA (2×10^7^ pfu) via tail vein injection of Sting^+/+^, Sting^Gt/Gt^, and IRF3^−/−^ mice. Serum was collected at 6 h post-infection. We found that MVA infection of Sting^+/+^ mice induces IFN-α and IFN-β production to the levels of 798 pg/ml and 1017 pg/ml, respectively, which was abolished in Sting^Gt/Gt^, and IRF3^−/−^ mice (Figure 4D). These results indicate that MVA-induced type I IFN production *in vivo* is also dependent on STING and transcription factor IRF3.

### cGAS is required for the induction of type I IFN by MVA in cDCs

**Figure 5:**
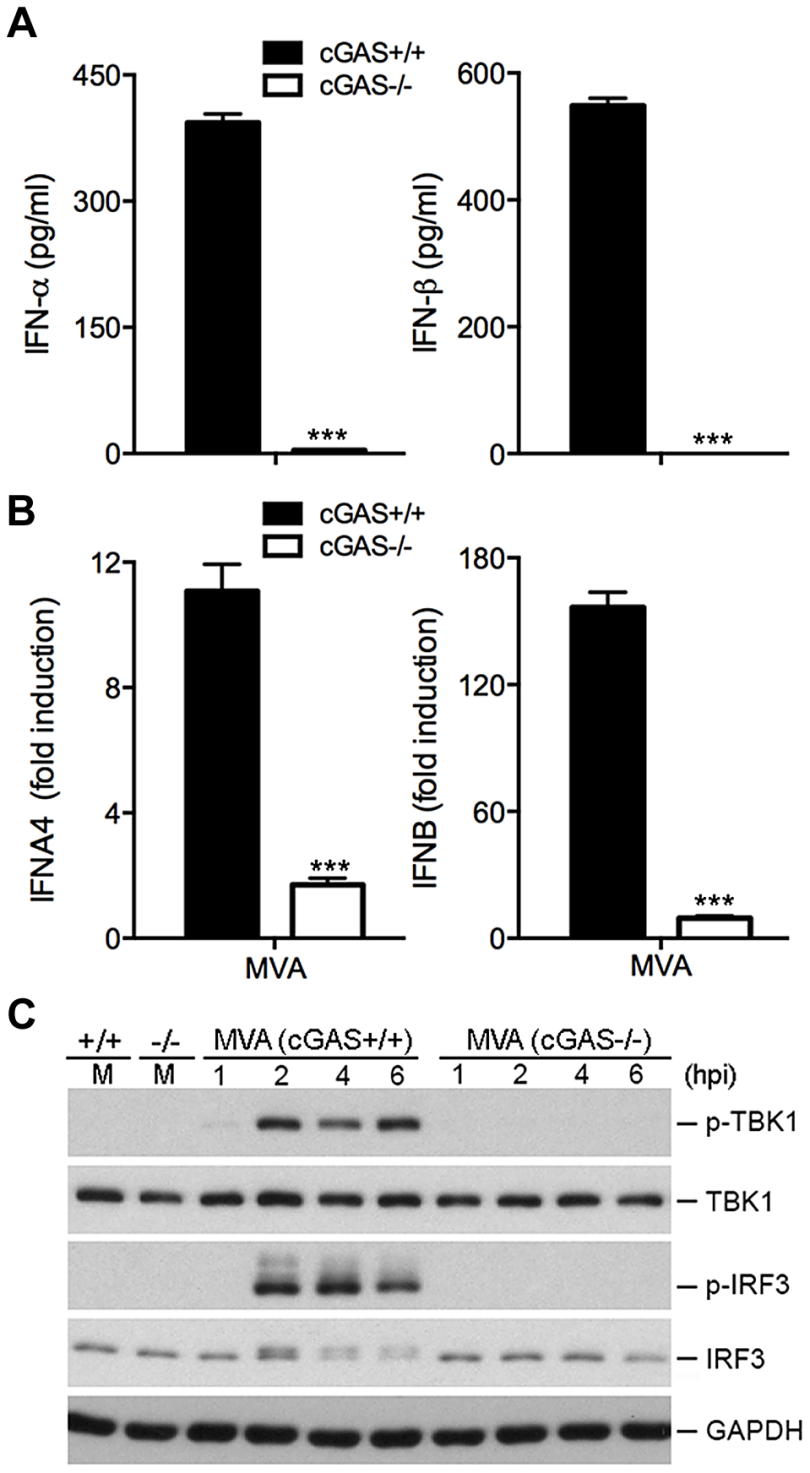
cGAS is the critical cytosolic DNA sensor for MVA infection of cDCs. GM-CSF-BMDCs were generated from cGAS^−/−^ mice and its age-matched WT controls. (A) Cells (1×10^6^) were infected with MVA at a MOI of 10. Supernatants were collected 22 h later. The concentrations of IFN-α and IFN-β were determined by ELISA. Data are means ± SEM (n = 3). A representative experiment is shown, repeated twice (***, *p*<0.001). (B) Cells (1×10^6^) were infected with MVA at a MOI of 10. Cells were collected at 6 h post infection. Real-time PCR analysis of IFNA4 and IFNB mRNAs were performed. Data are means ± SEM (n = 3). A representative experiment is shown, repeated twice (***, *p*<0.001). (C) Western blot analysis of cGAS^+/+^ and cGAS^−/−^ cDCs infected with MVA at a MOI of 10, or mock infected. Whole-cell lysates were prepared. Equal amount of proteins were subjected to SDS-PAGE and immunoblotting with anti-phospho-TBK1, anti-TBK1, anti-phosphoserine-396 of IRF3, and anti-IRF3. GAPDH was used as a loading control. “hpi”, hours post infection. “M”, mock infection control.

The STING/IRF3 pathway can be activated by cyclic GMP-AMP (cGAMP), a mammalian second messenger produced by cyclic GMP-AMP synthase (cGAS) in response to transfected DNA or DNA virus infection [56], [57]. Moreover, heat-inactivated vaccinia infection of THP1 (human acute monocytic leukemia cells) triggers the production of cGAMP. cGAS-deficient mice are more susceptible to herpes simplex virus 1 (HSV-1) infection than WT mice [58]. We generated cDCs from cGAS-deficient mice and WT controls. We found that MVA-induced IFN-α/β production was abolished in cGAS^−/−^ cells (Figure 5A). Induction of IFNA4 and IFNB mRNA by MVA was also diminished in cGAS^−/−^ cells compared with WT cells (Figure 5B). Western blot analysis demonstrated that MVA-induced phosphorylation of TBK1 and IRF3 was absent in cGAS^−/−^ cells (Figure 5C). These results established that cGAS is the critical cytosolic DNA sensor for MVA.

### Viral DNA replication is not required for MVA sensing in BMDCs

**Figure 6:**
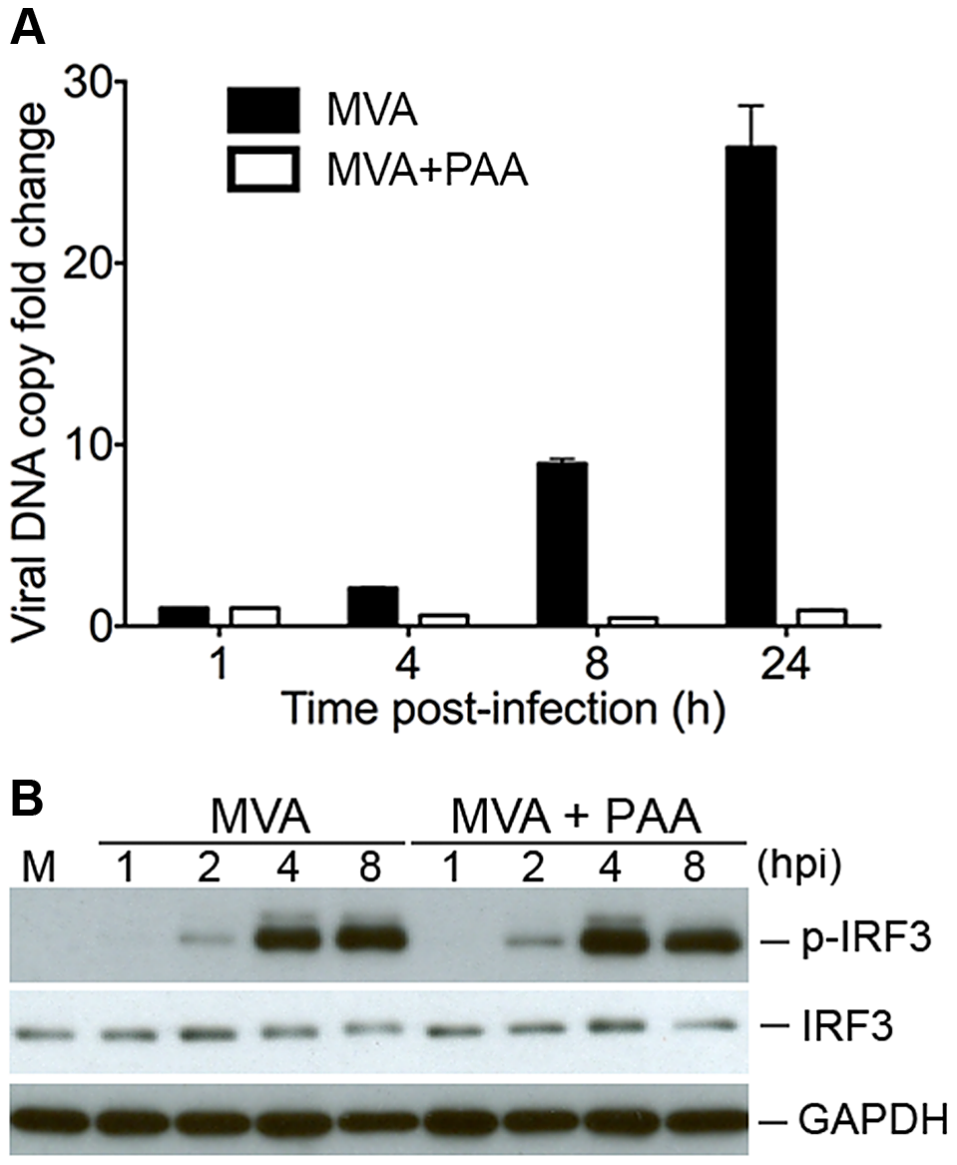
Viral DNA replication is not required for MVA-induced IRF3 phosphorylation. (A) GM-CSF-BMDCs (1×10^6^) were pre-incubated with either PAA at 200 µg/ml or with mock control for 1 h, and then infected with MVA at a MOI of 3 for 1 h. Cells were washed and incubated with fresh medium with or without PAA. Cells were collected at 1, 4, 8, and 24 h post infection. Viral DNA was extracted and purified. Real-time PCR was performed with primers and TagMan probe specific for the vaccinia ribonucleotide reductase l4L gene. (B) Western blot analysis of BMDCs infected with MVA at a MOI of 10 in the presence of absence of PAA. Whole-cell lysates were prepared. Equal amount of proteins were subjected to SDS-PAGE and immunoblotting with anti-phosphoserine-396 of IRF3 or anti-IRF3. Glyceraldehyde 3-Phosphate Dehydrogenase (GAPDH) was used as a loading control. “hpi”, hours post infection. “M”, mock infection control.

Using quantitative real-time PCR analysis, we found that WT vaccinia and MVA DNA genomic copies were increased by 392-fold and 111-fold, respectively, at 24 h post-infection of BSC40 cells, whereas DNA copies of the WT vaccinia and MVA were increased by 86-fold and 46-fold, respectively, at 24 h post infection of BMDCs (Figure S2). This result raised the question of whether viral DNA replication is required for type I IFN induction by MVA in cDCs. Phosphonoacetate (PAA) inhibits vaccinia DNA polymerase [59], [60]. Using real-time PCR analysis, we found that treatment of cDCs with PAA prevented MVA DNA replication (Figure 6A). Western blot analysis demonstrated that PAA treatment did not affect MVA-induced IRF3 phosphorylation (Figure 6B), indicating that viral DNA replication is not required for MVA-induced activation of STING/IRF3 pathway in cDCs.

### Type I IFN induction in murine cDCs by MVA requires endosomal and lysosomal acidification and lysosomal enzyme activity

**Figure 7:**
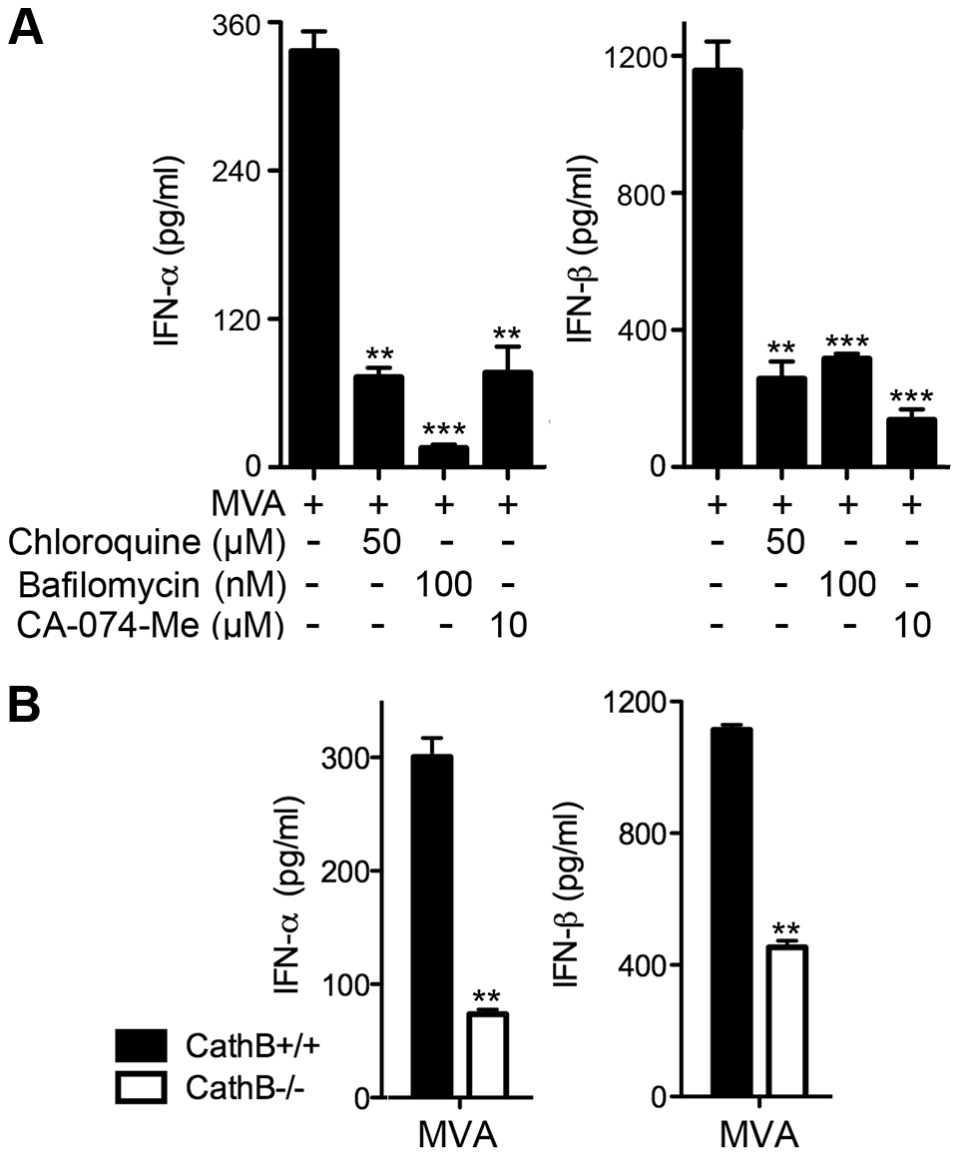
Endosomal and lysosomal acidification and lysosomal enzyme activities are required for type I IFN production by BMDCs in response to MVA infection. (A) GM-CSF-BMDCs (1×10^6^) were infected with MVA at a MOI of 10 for 1 h. Cells were washed and incubated in fresh medium in the presence or absence of chloroquine (50 µM), bafilomycin A1 (100 nM), or CA-074-Me (10 µM). Supernatants were collected 22 h later. The concentrations of IFN-α/β were determined by ELISA. Data are means ± SEM (n = 6). The combined results of three independently performed experiments are shown. **, *p*<0.01; ***, *p*<0.001; compared with no drug treatment. (B) GM-CSF-BMDCs were generated from Cathepsin B KO (CathB^−/−^) and age-matched WT controls (CathB^+/+^). Cells (1×10^6^) were infected with MVA at a MOI of 10. Supernatants were collected 22 h later. The concentrations of IFN-α/β were determined by ELISA. Data are means ± SEM (n = 3). A representative experiment is shown, repeated twice. **, *p*<0.01; comparisons were made between CathB^+/+^ and CathB^−/−^ cells as indicated.

To test whether lysosomal processing of virions might contribute to host sensing of MVA, we took both pharmacological and genetic approaches. Chloroquine and bafilomycin A1 block endosomal/lysosomal acidification and thereby may prevent virion processing in the late endosomes and lysosomes [61]. CA-074-Me is a specific inhibitor of cathepsin B, a lysosomal cysteine protease [62]. We infected cDCs with MVA at a MOI of 10. To avoid drug effects on viral entry, cells were treated at 1 h post infection with chloroquine at a final concentration of 50 µM, or bafilomycin A1 at 100 nM, or CA-074-Me at 10 µM. Supernatants were collected at 22 h post infection and measured for the concentrations of IFN-α/β by ELISA. We observed inhibition of IFN-α/β production in the presence of chloroquine, bafilomycin A1, and CA-074-Me (Figure 7A). These results suggest that MVA-mediated type I IFN induction requires endosomal/lysosomal processing of vaccinia virions. To test the role of cathepsin B in host sensing of MVA, we took advantage of the cathepsin B knock-out (KO) mice generated by targeted deletion of the *ctsb* gene [63]. We infected cDCs from cathepsin B^−/−^ (CathB^−/−^) mice and WT controls with MVA at a MOI of 10. Supernatants were collected at 22 h post infection. We found that IFN-α and IFN-β levels in the supernatant were reduced by 76% and 60%, respectively, in CathB^−/−^ cDCs compared to WT cells, indicating that lysosomal enzyme cathepsin B activity contributes to MVA sensing in cDCs (Figure 7B). It is possible that other cathepsins might also participate in the processing of virions in the endosomal/lysosomal compartments because type I IFN induction by MVA is not abolished in cathepsin B^−/−^ cDCs.

### MVA E3 dampens innate immune-sensing pathway

**Figure 8:**
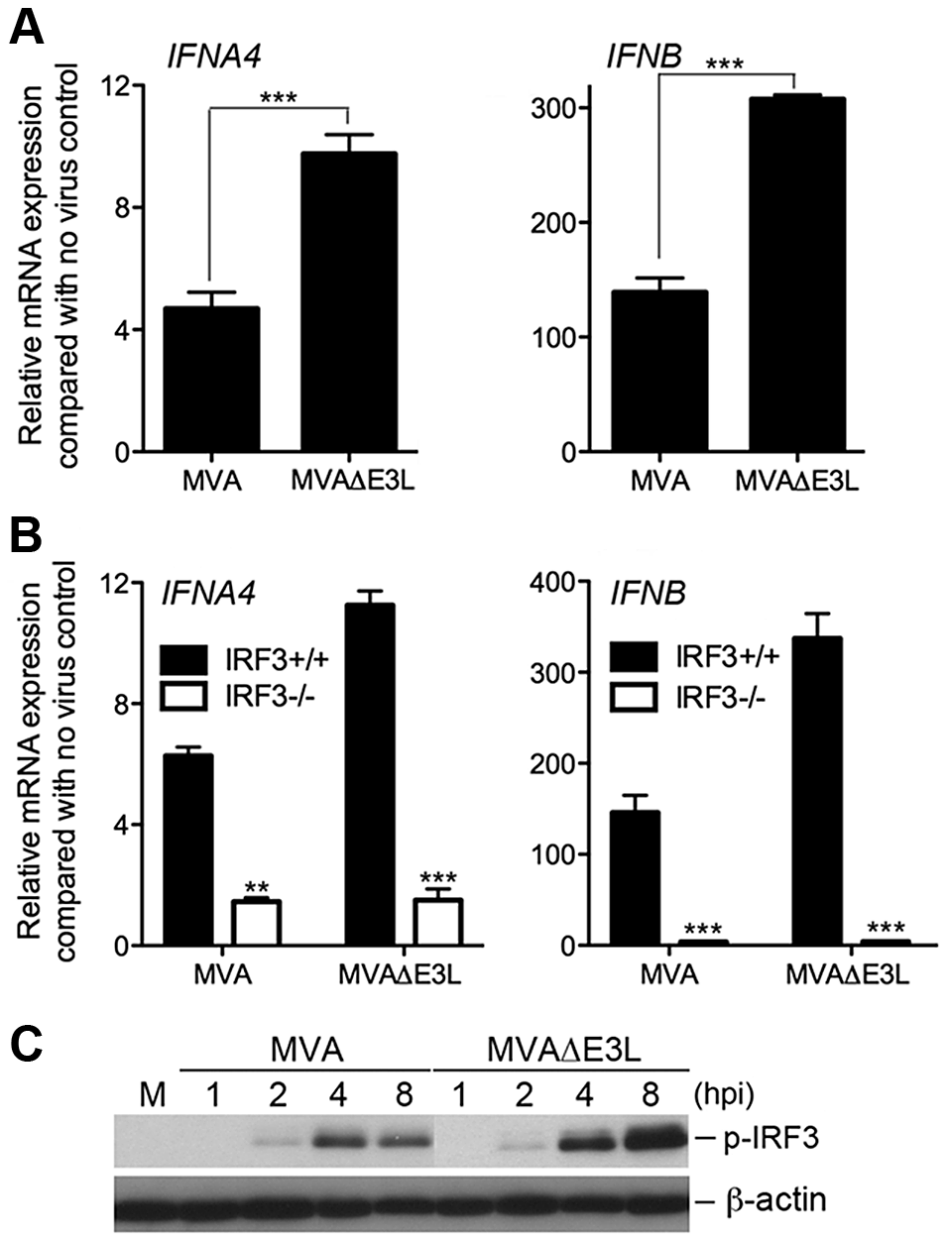
MVAΔE3L induces high levels of type I IFN gene expression in BMDCs than MVA does. (A) GM-CSF-BMDCs were generated from 6–8 week-old female WT C57B/6 mice. Cells (1×10^6^) were infected with MVA or MVAΔE3L at a MOI of 10. Cells were collected at 6 h post infection. Real-time PCR analysis of IFNA4 and IFNB mRNAs were performed. Data are means ± SEM (n = 3). A representative experiment is shown, repeated twice. ***, *p*<0.001; comparisons were made between MVA and MVAΔE3L infected cells. (B) GM-CSF-BMDCs were generated from IRF3^−/−^ mice and age-matched WT C57B/6 mice. Cells were infected with MVA or MVAΔE3L at a MOI of 10, or mock infected. Cells were collected at 6 h post infection. Real-time PCR analysis of IFNA4 and IFNB mRNAs were performed. Data are means ± SEM (n = 3). A representative experiment is shown, repeated twice. **, *p*<0.01; ***, *p*<0.001; comparisons were made between IRF3^+/+^ and IRF3^−/−^ cells. (C) Western blot analysis of GM-CSF-BMDCs infected with MVA or with MVAΔE3L at a MOI of 10, or mock infected. Whole-cell lysates were prepared. Equal amount of proteins were subjected to SDS-PAGE and immunoblotting with anti-phosphoserine-396 of IRF3. β-actin was used as a loading control. “hpi”, hours post infection. “M”, mock infection control.

E3 is a key virulence factor that attenuates various innate immune responses, including type I IFN induction. MVA retains the E3L gene. Western blot analysis showed that E3 protein was produced in WT VAC and MVA-infected BMDCs, but not in MVAΔE3L-infected cells (Figure S3). To test whether E3 plays an inhibitory role in MVA sensing in cDCs, we compared the induction of type I IFN gene expression between MVA and MVAΔE3L-infected cDCs. We found that infection with MVAΔE3L induced higher levels of IFNA4 and IFNB mRNAs than MVA (Figure 8A) (*p*<0.001). This induction was abolished in cells lacking transcription factor IRF3 (Figure 8B). Furthermore, Western blot analysis demonstrated that MVAΔE3L infection induced higher level of phospho-IRF3 than MVA at both 4 and 8 h post infection (Figure 8C). These results suggest that E3 dampens innate immune sensing of MVA and that removing E3 from MVA results in enhanced activation of type I IFN gene expression.

### Loss of a functional virulence factor N1 contributes to MVA-induced type I IFN induction and activation of TBK1 and IRF3 in cDCs

**Figure 9:**
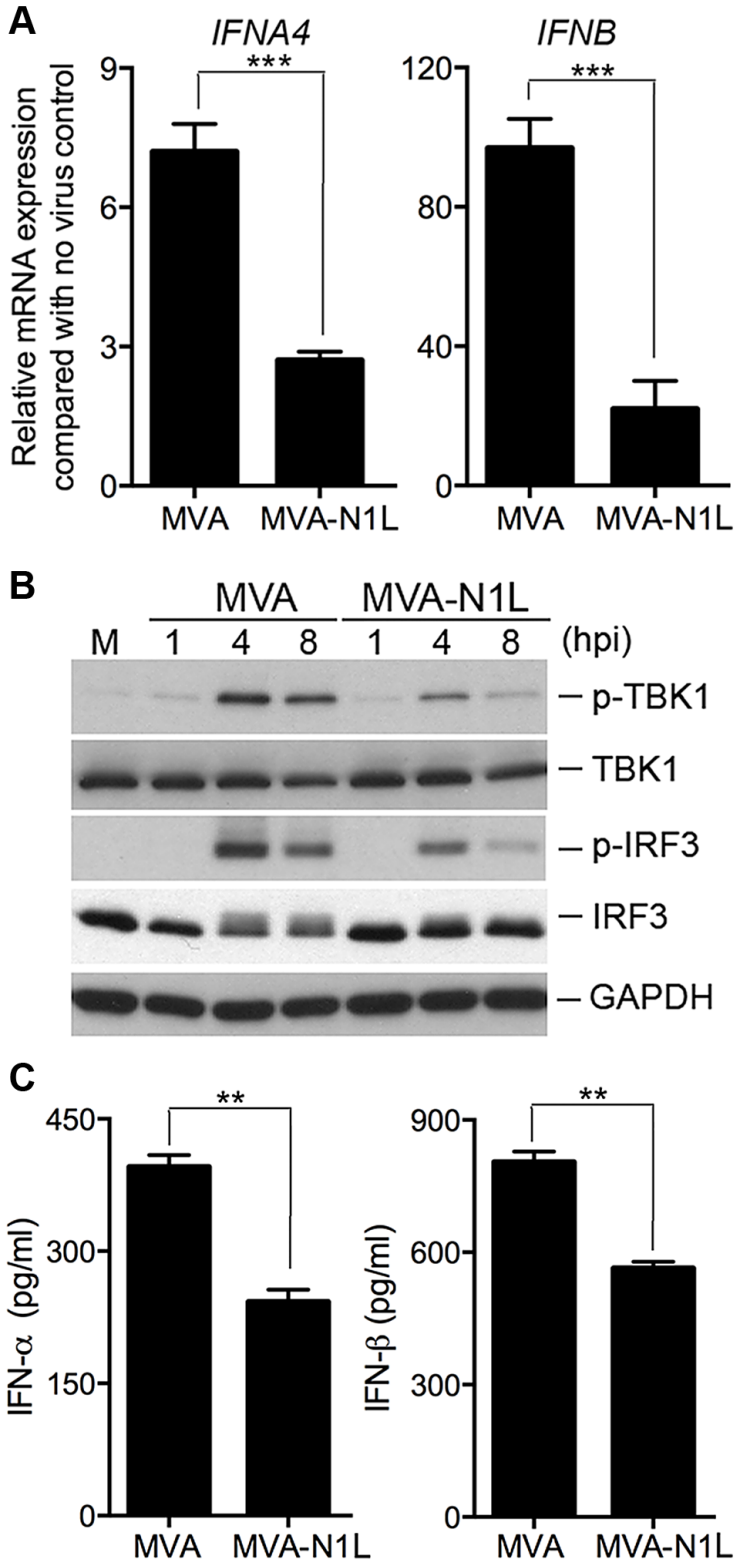
Vaccinia N1 virulence factor plays an inhibitory role in the type I IFN gene induction. GM-CSF-BMDCs (1×10^6^) were infected with MVA or MVA-N1L at a MOI of 10. (A) Cells were collected at 6 h post-infection. Real-time PCR analysis of IFNA4 and IFNB mRNAs were performed. Data are means ± SEM. A representative experiment is shown, repeated twice. (B) Western blot analysis of GM-CSF-BMDCs infected with MVA or MVA-N1L at a MOI of 10. Whole-cell lysates were prepared. Equal amount of proteins were subjected to SDS-PAGE and immunoblotting with anti-phospho-TBK1, anti-TBK1, anti-phosphoserine-396 of IRF3 and anti-IRF3. GAPDH was used as a loading control. “hpi”, hours post infection. “M”, mock infection control. (C) Cells (1×10^6^) were infected with MVA or MVA-N1L at a MOI of 10. Supernatants were collected 22 h later. The concentrations of IFN-α and IFN-β were determined by ELISA. Data are means ± SEM (n = 3). A representative experiment is shown, repeated twice (**, *p*<0.01).

Vaccinia virulence factor N1L gene is highly conserved among orthopoxviruses. The MVA orthologue of N1L contains a frameshift mutation resulting in a protein 4 amino acid (aa) shortened compared with N1 from WT VAC, but with a completely different C-terminal 23 aa segment [64], [65]. To test whether N1 alteration contributes to MVA-induction of the IFN pathway, we generated recombinant MVA that expresses the vaccinia N1L gene under its own promoter. Using Western blot analysis, we demonstrated that, unlike MVA, the recombinant virus MVA-N1L expresses vaccinia N1, which could be detected by a polyclonal antibody that recognizes the C-terminus of N1 (Figure S4). To investigate the role of N1 in modulating the IFN pathway, we infected cDCs with either MVA or MVA-N1L at a MOI of 10. Cells were harvested at 6 h post-infection and prepared for real-time PCR analysis for type I IFN mRNA expression. We found that the levels of IFNA4 and IFNB mRNAs in MVA-N1L-infected cDCs were 62% and 77% lower, respectively, compared with MVA-infected cells (Figure 9A) (*p*<0.001). Western blot analysis demonstrated that MVA-N1L infection triggered reduced levels of activation of p-TBK1 and p-IRF3 at 4 and 8 h post-infection when compared with MVA (Figure 9B). ELISA analysis of supernatants collected at 22 h post infection showed that MVA-N1-induced IFN-α and IFN-β levels were 39% and 30% lower, respectively, compared with those induced by MVA (Figure 9C) (*p*<0.01). Taken together, these data indicate that vaccinia N1 inhibits the cytosolic DNA-sensing pathway, which results in reduced type I IFN gene expression and secretion from cDCs.

## Discussion

Our study provides insights into how MVA infection is sensed by murine cDCs, a subset of DCs that are important for innate and adaptive antiviral immunity. We found that MVA induces type I IFN production in murine cDCs but not in pDCs, whereas WT vaccinia infection fails to induce type I IFN in either cDCs or pDCs. Using cDCs from knockout mice, we demonstrated that MVA-induced type I IFN induction is dependent on the newly discovered cytosolic DNA sensor cGAS and its adaptor, STING. The transcription factors IRF3 and IRF7, and the type I IFN feedback loop mediated by IFNAR1 are critical for the induction. By contrast, the TLR9/MyD88 pathway plays a relatively minor role in the sensing of MVA in cDCs. Treatment with inhibitors of either endosomal/lysosomal acidification or lysosomal enzyme activity attenuated MVA-induced type I IFN production, indicating that lysosomal enzymatic processing of vaccinia virions is important for MVA sensing in cDCs. Finally, we showed that vaccinia virulence factors E3 and N1 play inhibitory roles in IFN-β induction.

Based on these results, we hypothesize that following viral entry, possibly through macropinocytosis, some of the MVA virions are processed in the late endosomal and lysosomal compartments. As a result, viral DNAs are released into the cytoplasm and then sensed by the cytosolic DNA sensor cGAS, which leads to production of cGAMP [56], [58], [66]–[69]. cGAMP then acts as a ligand for STING to trigger the recruitment and activation of TBK1, which results in the phosphorylation of IRF3, as well as the subsequent induction of type I IFN gene expression [57], [70]–[72]. We surmise that viral DNA is the primary stimulator for type I IFN induction in response to MVA infection. We conclude that neither the endosomal dsRNA-sensing pathway (mediated by TLR3/TRIF) nor the cytosolic dsRNA-sensing pathway (mediated by MDA5/MAVS) is involved in MVA-induced type I IFN production.

STING has been identified as a critical adaptor for the cytosolic DNA sensor(s) [49]–[53]. It also acts as a direct sensor for cyclic dinucleotides, a type of bacterial second messenger [73]. STING is important for host defense against DNA viruses [50], [74]. Besides cGAS, several other cytosolic DNA sensors utilizing STING as an adaptor, including IFI16 and DDX41, have been identified [75], [76]. We found that shRNA knock-down of DDX41 in macrophage cell line failed to reduce MVA-induced phosphorylation of TBK1 and IRF3 (data not shown). We were unable to detect any difference of MVA-induced type I IFN production in IFI16-deficient cDCs compared with WT cells (data not shown).

Transcription factors IRF3 and IRF7 play important roles in the induction of type I IFN genes and the activation of antiviral viral immunity [77]. Our results indicate that IRF3 is required for the induction of IFN-α/β secretion by MVA-infected cDCs, whereas IRF7 also contributes to the initial induction of IFN-β production and the subsequent amplification of the type I IFN signal and induction of IFN-α production. Daffis et al. reported that West Nile Virus (WNV)-induced IFNB gene expression and that IFN-β production in cDCs is independent of IRF3 and IRF7 [78]. This could be due to different upstream sensing mechanisms for MVA and WNV in cDCs.

Vaccinia E3, a virulence factor, inhibits multiple signaling pathways that are important in host antiviral responses. E3 is a 190-aa protein that is composed of two distinct domains, the N-terminal Z-DNA binding domain (ZBD) and the C-terminal dsRNA binding domain (dsRBD). MVA retains the E3L gene, which is expressed in MVA-infected cells. MVAΔE3L infection of cDCs triggers higher gene expression of type I IFN than MVA, but no significant differences in type I IFN secretion was observed between MVA and MVAΔE3L-infected cDCs (Dai and Deng, unpublished). Others have shown that MVAΔE3L induces a higher level of apoptosis than MVA in HeLa cells and MEFs [79]–[81]. Consistent with published results, we found that MVAΔE3L infection of cDCs induces more apoptosis of infected cells than MVA.

How vaccinia E3 inhibits the cytosolic DNA-sensing pathway is currently unclear. It might prevent the activation of cGAS via its ZBD. Alternatively, it may block the activation of its downstream effector(s). We have previously shown that E3 ZBD plays an inhibitory role in TLR9/MyD88/IRF7-mediated myxoma virus-sensing in pDCs [17], whereas E3 dsRBD attenuated the cytosolic dsRNA-sensing pathway mediated by MAVS/IRF3 [35]. It has been recently shown that ZBD of E3 antagonizes PKR in primary MEF [82]. The present study illustrates another inhibitory function of E3 ZBD in the cytosolic DNA-sensing pathway. In fact, E3LΔ83N, a mutant vaccinia virus lacking the ZBD, is attenuated in intranasal and intracranial infection models [27], [31], further supporting the *in vivo* function of the ZBD domain of E3.

Vaccinia N1, another virulence factor, is altered in MVA due to a frameshift mutation, which results in a shortened polypeptide with a complete different 23-aa at the C-terminus. To test whether N1 inhibits the STING-dependent cytosolic DNA-sensing pathway, we generated a recombinant MVA that expresses the vaccinia N1L gene under its natural promoter. We found that infection with MVA-N1L virus induces lower levels of IFNA4 and IFNB mRNAs, as well as lower levels of p-TBK1 and p-IRF3. N1 has been shown to be able to interact with TBK1 [21]. We conclude that vaccinia N1 is an inhibitor of the STING-dependent type I IFN pathway, possibly through its interaction with TBK1.

Delaloye et al. [38] reported that MVA infection of human THP-1 cells induced type I IFN that was dependent on the cytosolic dsRNA-sensing pathway mediated by MDA5 and IPS-1/MAVS using shRNA knockdown. However, the cytosolic DNA-sensing pathway was not assessed in that paper. Using BMDCs from MDA5 or MAVS KO mice, we found that the cytosolic dsRNA- sensing pathway mediated by MDA5/MAVS was not required for type I IFN induction by MVA in these cells. This might be due to the intrinsic differences between the human and murine system and different experimental approaches. Future studies are needed to elucidate the induction of type I IFN in human DCs, which has implications for the use of MVA in vaccinations.

Waibler et al. [37] reported that MVA infection of bone marrow-derived Flt3-L cultured DCs or bone marrow-derived GM-CSF cultured DCs induced type I IFN induction, which was primarily triggered by non-TLR sensors and was independent of viral replication. At the time of the investigation, cytosolic DNA sensors had not been discovered. In our present study, we took advantage of FACS sorting to generate highly purified pDC and cDCs populations that allow us to demonstrate that MVA-induction of type I IFN mainly occurs in cDCs, but not in pDCs. Using STING-deficient BMDCs, we were able to show that STING plays a critical role in sensing MVA infection in cDCs.

Our present study and previous reports on host innate immune sensing of MVA and myxoma virus reveal some key differences between the two poxviruses. Whereas MVA is a strong inducer of type I IFN in cDCs via the activation of the cytosolic DNA-sensing pathway mediated by cGAS/STING/IRF3, it fails to induce IFN in pDCs. By contrast, myxoma infection of pDCs potently induces type I IFN through the endosomal DNA-sensing pathway mediated by TLR9/MyD88/IRF7 [17], [18], whereas myxoma infection of cDCs fails to activate IRF3 or to induce type I IFN (Dai and Deng, unpublished). Although MVA has been studied intensively as a vaccine vector, the studies have not been conducted with myxoma virus backbone and should be done in the future.

In summary, we present data demonstrating the critical role of the cGAS/STING pathway in mediating MVA-induced type I IFN production in murine cDCs. We postulate that upon viral entry, some of the MVA virions are processed in the endosomal/lysosomal compartment, and viral DNAs are detected by the cytosolic DNA sensor(s), leading to the assembly of the STING complex and activation of transcription factors IRF3 and IRF7. We provide evidence that vaccinia E3 and N1 play inhibitory roles in this pathway. Our future studies will focus on the mechanisms by which E3 and N1 attenuate the cGAS/STING-mediated cytosolic DNA-sensing pathway, as well as the role of cGAS and STING in host defense against vaccinia infection and MVA-induced adaptive immunity *in vivo*. The results of these studies will provide insights into improved poxvirus-based vaccines for clinical applications for both infectious diseases and cancers.

## Materials and Methods

### Ethics statement

Mice were maintained in the animal facility at the Sloan Kettering Institute. All procedures were performed in strict accordance with the recommendations in the Guide for the Care and Use of Laboratory Animals of the National Institute of Health.

### Viruses and cell lines

The WR strain of vaccinia virus was propagated and virus titers were determined on BSC40 (African green monkey kidney cells) monolayers at 37°C. MVA and MVAΔE3L viruses were kindly provided by Gerd Sutter (University of Munich), and propagated in BHK-21 (baby hamster kidney) cells. MVA-N1L recombinant virus was generated by using a two-step, red-mediated recombination system [83]. The MVA orthologue of N1L containing a truncated and mutated C terminus of 71 nucleotides (due to a frameshift) was reconstituted by orthotopic insertion of 83 nucleotides of VACV N1L, which resulted in a full-length N1L gene. MVA-N1L was propagated on chicken embryonic fibroblasts (CEFs). All of the viruses were purified through a 36% sucrose cushion. BSC40 cells were maintained in Dulbecco's modified Eagle's medium (DMEM) supplemented with 5% fetal bovine serum (FBS). BHK-21 and RK13 cells were cultured in DMEM containing 10% FBS, 0.1 mM nonessential amino acids, and 50 µg/ml gentamycin. All cells were grown at 37°C in a 5% CO_2_ incubator.

### Mice

Female C57B/6 mice between 6 and 10 weeks of age were purchased from the Jackson Laboratory and were used for the preparation of bone marrow-derived dendritic cells. These mice were maintained in the animal facility at the Sloan Kettering Institute. All procedures were performed in strict accordance with the recommendations in the Guide for the Care and Use of Laboratory Animals of the National Institute of Health. The protocol was approved by the Committee on the Ethics of Animal Experiments of Sloan-Kettering Cancer Institute. cGAS^−/−^, MDA5^−/−^, MAVS^−/−^, IRF3^−/−^, IRF7^−/−^, TLR3^−/−^, MyD88^−/−^, TLR9^−/−^, TLR7^−/−^, TRIF^−/−^ (TRIF^LPS2/LPS2^), Cathepsin B^−/−^, and STING^Gt/Gt^ mice were generated in the laboratories of Drs. Zhijian Chen (University of Texas Southwestern Medical Center), Marco Colona (Washington University), Tadatsugu Taniguchi (University of Tokyo), Richard Flavell (Yale University), Shizuro Akira (Osaka University), Bruce Beutler (Scripps Research Institute), Christoph Peters (University of Freiburg), and Russell Vance (University of California, Berkeley). IFNAR1^−/−^ mice were provided by Dr. Eric Pamer (Sloan Kettering Institute); the mice were purchased from B&K Universal and were backcrossed with C57BL/6 mice for more than five generations.

### Generation of bone marrow-derived dendritic cells

The bone marrow cells from the tibia and femur of mice were collected by first removing muscles from the bones, and then flushing the cells out using 0.5 cc U-100 insulin syringes (Becton Dickinson) with RPMI with 10% FCS. After centrifugation, cells were re-suspended in ACK Lysing Buffer (Lonza) for red blood cells lysis by incubating the cells on ice for 1–3 min. Cells were then collected, re-suspended in fresh medium, and filtered through a 40-µm cell strainer (BD Biosciences). The number of cells was counted. For the generation of Flt3L-BMDCs, the bone marrow cells (5 million cells in each well of 6-well plates) were cultured in complete medium (CM) in the presence of Flt3L (100 ng/ml; R & D systems) for 7–9 days. Cells were fed every 2–3 days by replacing 50% of the old medium with fresh medium. FACS was used to obtain highly purified pDCs and cDCs. pDCs were gated as CD11C^+^B220^+^PDCA-1^+^ and cDCs were gated as CD11C^+^B220^−^PDCA-1^−^ as described [17]. For the generation of GM-CSF-BMDCs, the bone marrow cells (5 million cells in each 10 cm cell culture dish) were cultured in CM in the presence of GM-CSF (30 ng/ml, produced by the Monoclonal Antibody Core facility at the Sloan Kettering Institute) for 10–12 days. CM is RPMI 1640 medium supplemented with 10% heat-inactivated fetal bovine serum (FBS), 100 U/ml penicillin, 100 µg/ml streptomycin, 0.1 mM essential and nonessential amino acids, 2 mM L-glutamine, 1 mM sodium pyruvate, and 10 mM HEPES buffer. Cells were fed every 2 days by replacing 50% of the old medium with fresh medium and re-plated every 3–4 days to remove adherent cells. Only non-adherent cells were used for experiments.

### RNA isolation and real-time PCR

RNA was extracted from whole-cell lystates with a RNeasy Mini kit (Qiagen) and was reverse transcribed with a First Strand cDNA synthesis kit (Fermentas). Quantitative real-time PCR was performed in triplicate with SYBR Green PCR Mater Mix and Applied Biosystems 7500 Real-time PCR Instrument (Life Technologies) using gene-specific primers. Relative expression was normalized to the levels of glyceraldehyde-3-phosphate dehydrogenase (GADPH).

### Cytokine assays

Cells were infected with various viruses at a MOI of 10 for 1 h or mock infected. The inoculum was removed and the cells were washed with PBS twice and incubated with fresh medium. Supernatants were collected at various times post infection. Cytokine levels were measured by using enzyme-linked immunosorbent essay (ELISA) kits for IFN-α/β (PBL Biomedical Laboratories) and CCL5 (R & D systems).

### Western blot analysis

BMDCs (1×10^6^) were infected with MVA at a MOI of 10. At various times post-infection, the medium was removed and cells were collected. Whole-cell lysates were prepared at 1, 2, 4, 6, and 9 h post-infection. Equal amounts of proteins were subjected to sodium dodecyl sulfate-polyacrylamide gel electrophoresis and the polypeptides were transferred to a nitrocellulose membrane. Phosphorylation of IRF3 was determined using a rabbit polyclonal antibody specific for phosphoserine-396 of IRF3 (Cell Signaling). The level of IRF3 was determined by using a rabbit polyclonal antibody against IRF3. Anti-phospho-TBK1, anti-TBK1 and anti-STING antibodies were purchased from Cell Signaling. Vaccinia E3 protein level was determined by using anti-E3 monoclonal antibody (MAb 3015B2) kindly provided by Dr. Stuart N. Isaacs (University of Pennsylvania) [84]. Vaccinia N1 protein level was assessed by using mouse monoclonal anti-N1 antibody (7E5) kindly provided by Dr. Michael Way (Cancer Research UK). Vaccinia H3 protein level was assessed by using rabbit polyclonal anti-H3 antibody (a kind gift from Bernard Moss, NIH). Anti-glyceraldehyde-3-phosphate dehydrogenase (GADPH) or anti-β-actin antibodies were used as loading controls.

### Viral DNA copy number analysis by real-time PCR

Viral DNA was purified with the DNeasy Mini Kit (Qiagen) according to the manufacture's protocol. Real-time PCR was performed with the Applied Biosystems 7500 Real-Time PCR Instrument (Life Technologies). The primers and TaqMan probe used in the quantitative PCR assay are specific for the vaccinia ribonucleotide reductase l4L gene. The sequences and PCR condition were described by Liu et.al. [85]. A standard curve was established from cloned DNA fragment of the vaccinia l4L gene. Corresponding CT (cycle threshold) values obtained by the real-time PCR methods were plotted on the standard curve to calculate the viral DNA copy number.

### Reagents

The commercial sources for reagents were as follows: CpG oligodeoxynucleotide ODN2216 (Invivogen); chloroquine, bafilomycin A1 and CA-074-Me (Sigma-Aldrich); Flt3L (R & D systems); anti-mouse CD11c APC (BD Pharmingen), anti-mouse B220 APC-Cy7 and anti-mouse PDCA-1 PE (Milteny Biotec); and Phosphonoacetate (Sigma).

### Statistics

Student's two-tailed *t*-test was used for each pairwise comparison. The p values deemed significant are indicated in the figures as follows: *, *p*<0.05; **, *p*<0.01; ***, *p*<0.001.

## Supporting Information

Figure S1 **TLR3/TRIF and MDA5/MAVS are not required for the induction of type I IFN in cDCs by MVA.** GM-CSF-BMDCs were generated from TLR3^−/−^ (A), TRIF^−/−^ (B), MDA5^−/−^ (C), MAVS^−/−^ mice (D) and their age-matched WT controls. Cells (1×10^6^) were either stimulated with CpG or infected with MVA at a MOI of 10. Supernatants were collected 22 h later. The concentrations of IFN-α and IFN-β were determined by ELISA. Data are means ± SEM (n = 3). A representative experiment is shown, repeated once.
(TIF)

Figure S2 **Viral DNA replication in BSC40 and BMDCs.** BSC40 cells and GM-CSF-BMDCs (1×10^6^) were infected with WT VAC or MVA at a MOI of 3 for 1 h. Cells were washed and incubated with fresh medium with or without PAA. Cells were collected at 1, 4, 8, and 24 h post inoculation. Viral DNA was extracted and purified. Real-time PCR was performed with primers and TagMan probe specific for the vaccinia ribonucleotide reductase l4L gene.
(TIF)

Figure S3 **Expression of E3 by WT vaccinia and MVA infected BMDCs.** Western blot analysis of GM-CSF-BMDCs infected with WT VAC, MVA, or MVAΔE3L at a MOI of 10, or mock infected. Whole-cell lysates were prepared. Equal amount of proteins were subjected to SDS-PAGE and immunoblotting with anti-E3 monoclonal antibody. β-actin was used as a loading control. “hpi”, hours post infection. “M”, mock infection control.
(TIF)

Figure S4 **Expression of N1 by WT vaccinia and MVA-N1L viruses.** (A) HeLa cells (2×10^6^) were infected with WT VAC, ΔN1L, MVA, or MVA-N1L at a MOI of 5, or mock infected. Cells were collected at 24 h post-infection. Western blot analysis was performed using antibodies to vaccinia H3 and N1. (B) BMDC (2×10^6^) were infected with WT VAC, MVA-N1L, or MVA at a MOI of 20, or mock infected. At various time points post infection, medium was removed and cells were collected. Western blot analysis was performed. Vaccinia virus proteins N1 or H3 were detected by mouse monoclonal anti-N1 (7E5) or rabbit polyclonal anti-H3, respectively. β-actin was used as a loading control. “hpi”, hours post infection.
(TIF)
