# Supplementary figures and images for: Modified Vaccinia Virus Ankara Triggers Type I IFN Production in Murine Conventional Dendritic Cells via a cGAS/STING-Mediated Cytosolic DNA-Sensing Pathway
Source: PLoS Pathog. 2014 Apr 17;10(4):e1003989. doi: 10.1371/journal.ppat.1003989 (PMC3990710; doi:10.1371/journal.ppat.1003989)

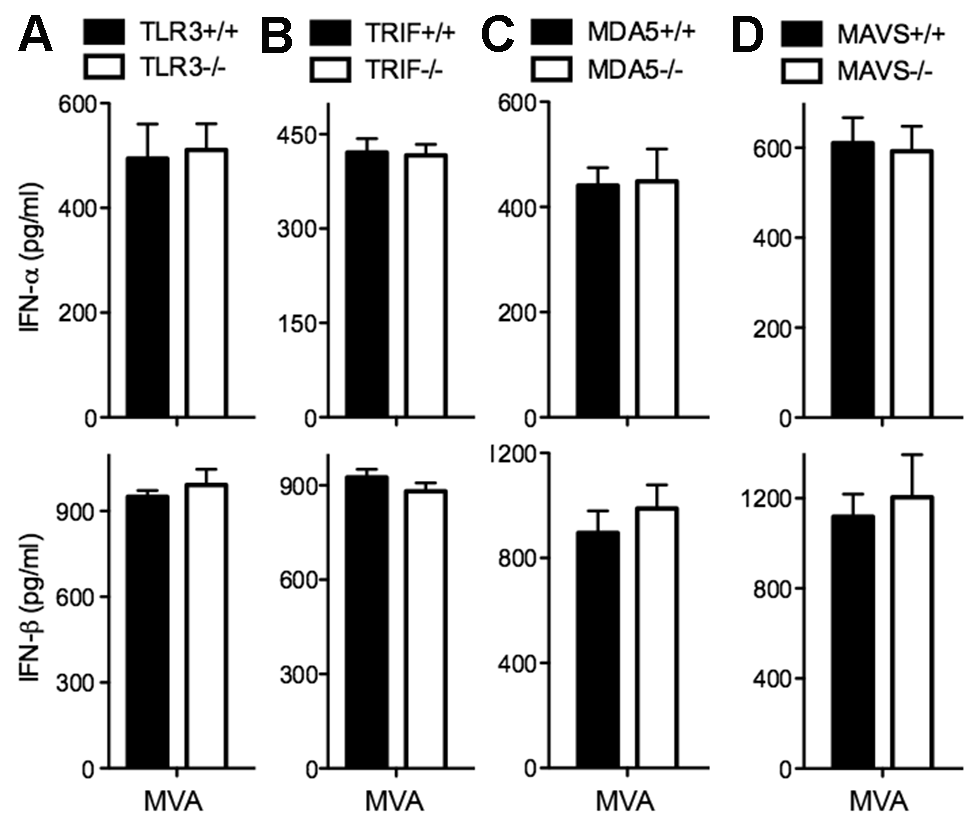

Supplement: Figure S1 — TLR3/TRIF and MDA5/MAVS are not required for the induction of type I IFN in cDCs by MVA. GM-CSF-BMDCs were generated from TLR3−/− (A), TRIF−/− (B), MDA5−/− (C), MAVS−/− mice (D) and their age-matched WT controls. Cells (1×10^6^) were either stimulated with CpG or infected with MVA at a MOI of 10. Supernatants were collected 22 h later. The concentrations of IFN-α and IFN-β were determined by ELISA. Data are means ± SEM (n = 3). A representative experiment is shown, repeated once. (TIF) [file ppat.1003989.s001.tif]

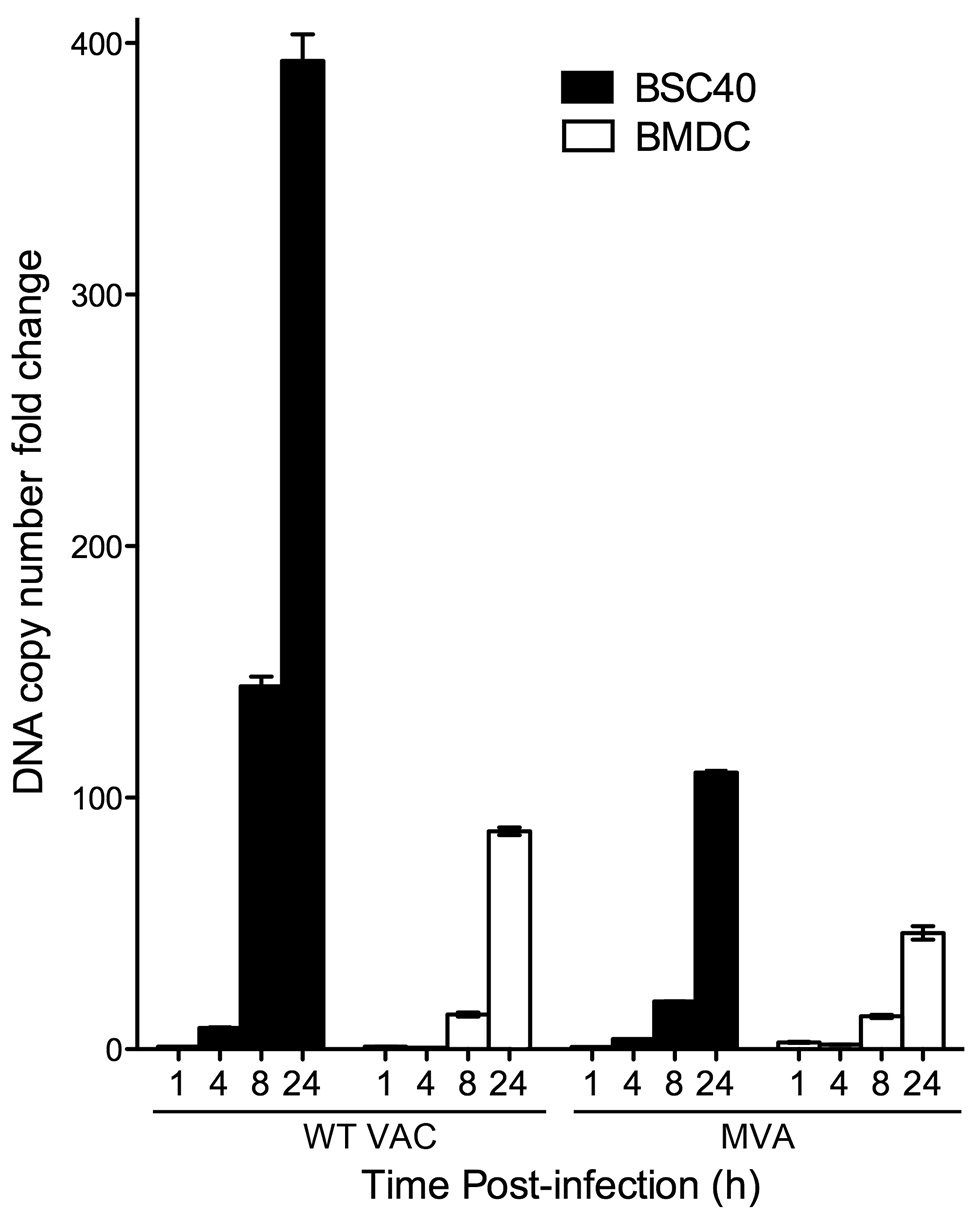

Supplement: Figure S2 — Viral DNA replication in BSC40 and BMDCs. BSC40 cells and GM-CSF-BMDCs (1×10^6^) were infected with WT VAC or MVA at a MOI of 3 for 1 h. Cells were washed and incubated with fresh medium with or without PAA. Cells were collected at 1, 4, 8, and 24 h post inoculation. Viral DNA was extracted and purified. Real-time PCR was performed with primers and TagMan probe specific for the vaccinia ribonucleotide reductase l4L gene. (TIF) [file ppat.1003989.s002.tif]

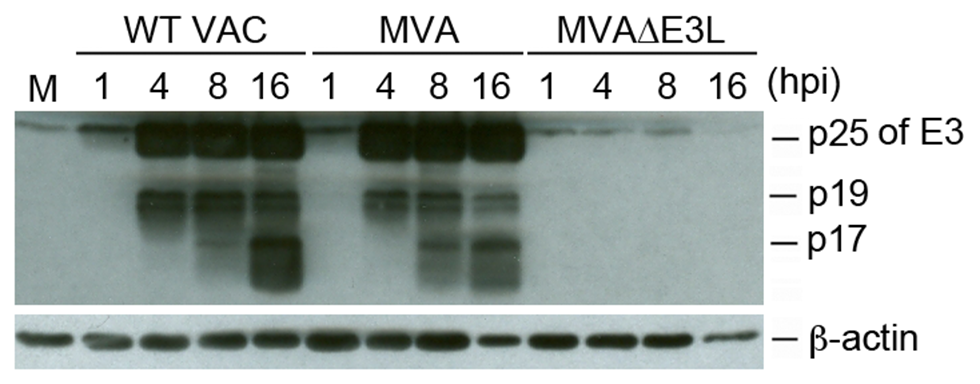

Supplement: Figure S3 — Expression of E3 by WT vaccinia and MVA infected BMDCs. Western blot analysis of GM-CSF-BMDCs infected with WT VAC, MVA, or MVAΔE3L at a MOI of 10, or mock infected. Whole-cell lysates were prepared. Equal amount of proteins were subjected to SDS-PAGE and immunoblotting with anti-E3 monoclonal antibody. β-actin was used as a loading control. “hpi”, hours post infection. “M”, mock infection control. (TIF) [file ppat.1003989.s003.tif]

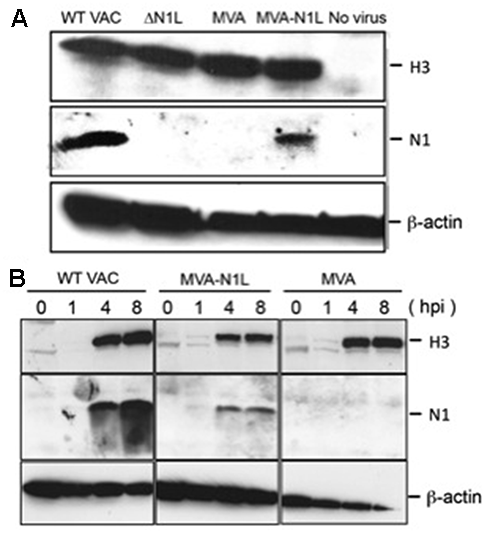

Supplement: Figure S4 — Expression of N1 by WT vaccinia and MVA-N1L viruses. (A) HeLa cells (2×10^6^) were infected with WT VAC, ΔN1L, MVA, or MVA-N1L at a MOI of 5, or mock infected. Cells were collected at 24 h post-infection. Western blot analysis was performed using antibodies to vaccinia H3 and N1. (B) BMDC (2×10^6^) were infected with WT VAC, MVA-N1L, or MVA at a MOI of 20, or mock infected. At various time points post infection, medium was removed and cells were collected. Western blot analysis was performed. Vaccinia virus proteins N1 or H3 were detected by mouse monoclonal anti-N1 (7E5) or rabbit polyclonal anti-H3, respectively. β-actin was used as a loading control. “hpi”, hours post infection. (TIF) [file ppat.1003989.s004.tif]
